# Role of Parental Attachment Styles in Moderating Interaction Between Parenting Stress and Perceived Infant Characteristics

**DOI:** 10.3389/fpsyg.2021.730086

**Published:** 2021-10-20

**Authors:** Maria Quintigliano, Cristina Trentini, Alexandro Fortunato, Marco Lauriola, Anna Maria Speranza

**Affiliations:** ^1^Department of Dynamic and Clinical Psychology, and Health Studies, Sapienza University of Rome, Rome, Italy; ^2^Department of Social and Developmental Psychology, Sapienza University of Rome, Rome, Italy

**Keywords:** infancy, parenting stress, attachment styles, social communication abilities, sensory regulatory functions

## Abstract

By employing the transactional model of development and focusing on the multifactorial nature of parenting, this study aimed to (1) examine whether important risk factors, particularly mothers’ insecure attachment styles and parenting stress contribute to the perception of their infants’ characteristics and (2) explore whether maternal attachment styles moderate the relationship between parenting stress and perceived infants’ characteristics. We recruited 357 mothers (age: 34.23; ± 5.38) who had 1-year-old infants (161 males and 196 females; age: 12.70; ± 1.60 months). All the mothers completed three self-report instruments: Parenting Stress Index–Short Form (PSI-SF), Attachment Style Questionnaire (ASQ), and 1st-Year Inventory (FYI). Although the latter was originally developed to determine the risk for autism in 1-year-olds, it was employed in this study to measure infant’s characteristics within two domains: social communication and sensory regulatory functions. Multiple regression analyses revealed that one of the PSI-SF dimensions - specifically the Parent–Child Dysfunctional Interaction - contributed to mothers’ perceptions of their children’s social communication abilities, whereas the attachment style did not. Other multiple regression analyses showed that all the dimensions of parenting stress - that is, Parenting Distress (PD), Parent-Child Dysfunctional Interaction (PCDI), and Difficult Child (DC) - contributed to mothers’ perceptions of their sensory regulatory abilities. The attachment styles, particularly anxious attachment, contributed significantly to a biased perception of these abilities controlled for parenting stress. Mothers reporting high levels of avoidance and high levels of PD viewed their children as less able in the social communicative domain (SC Dom) than if they had low levels of PD. By contrast, when levels of avoidance were low, mothers with high PD perceived their children as less difficult in the SC Dom than those with low levels of PD. Moreover, high avoidance levels influenced how mothers who considered the interaction with their children as difficult perceived them as having greater difficulties in relation to sensory regulatory domain (SR Dom). By contrast, mothers with high levels of anxiety high levels of PD view their children as less able in the SC Dom than if they had low levels of PD. When mothers’ levels of anxiety were very low, those with high PD viewed their children as less difficult in the SC Dom in comparison to those with low levels of PD.

## Introduction

Early affective relationships are widely considered crucial in child development. The transactional model of development (Sameroff and Chandler, 1975) considers the dynamic interchange between genetic, constitutional, neurobiological, biochemical, psychological, and sociological factors in the determination of behavior. The model further posits that the manifestation of psychopathology is dependent not only on environmental support but also on children’s characteristics, which, in turn, have a partial effect on the nature of the environment (Leslie and Cicchetti, 2016).

Sroufe and Rutter (1984) and Cicchetti et al. (1995) have conceived parenting as a multifactorial process, wherein the intersubjective dimension therefore plays a relevant role. Belsky (1984) proposed a model of parenting, which includes three primary determinants, specifically: social environment, parents’ personality, and infants’ characteristics. This perspective holds that parents and their children are active agents who, by virtue of continual transactions, cocreate their relationship (Maccoby, 1994; Collins et al., 2000). Coherently, research has largely documented that parent–child relationship involves more than the sum of individual contributions (Sroufe and Rutter, 1984; Cicchetti et al., 1995; Emde, 2007; Speranza et al., 2020).

As regards the children’s contribution to this process, it is noteworthy that infants have their specific ways of communicating, interacting, and regulating their emotional states. Speranza et al. (2020) noted that withdrawal and anxiety are two of such emotional states. These specificities enable parents to understand and manage their children. One-year-old infants, for example, already demonstrate a set of abilities – including social orienting, receptive communication, social-affective engagement, imitation, expressive language, and specific self-regulatory abilities (Baranek et al., 2003) – to which the parents need to adapt.

### Attachment and Parenting

Other than specific parents’ characteristics, such as intrusiveness, detachment, and psychological difficulties (Speranza et al., 2020), attachment style is one of the most important factors that can influence parenting. Accordingly, Bowlby (1969, 1988) proposed that caregiving results from an organized behavioral system, namely the attachment system, with which it evolves in parallel (George and Solomon, 1996, 1999). The primary aim of the caregiving system is to promote proximity and comfort when mothers perceive that their children are in real or potential danger. Mothers’ ability to regulate their children’s feelings of security is sustained by maternal sensitivity, which involves understanding their children’s feelings and responding to them appropriately (Ainsworth, 1967, 1973; Ainsworth et al., 1978).

The internal working models remain fairly stable across the lifespan, affecting individuals’ functioning, particularly in the construction of significant relationships (Bowlby, 1988; Shaver and Mikulincer, 2002; Cassidy and Shaver, 2008). Several studies have demonstrated the importance of adult attachment in parenting behavior, romantic relationships, as well as in mental and physical health (Bretherton, 1985; Ainsworth, 1989; Cassidy et al., 2013; Mikulincer and Shaver, 2016).

The proliferation of studies on adult attachment is partly due to the development of self-report instruments (such as the Attachment Style Questionnaire - ASQ; Feeney et al., 1994), which have been proved to reliably assess typical thoughts, feelings, and behaviors in the context of close relationships (Shaver and Mikulincer, 2004).

Hazan and Shaver (1987), who may be considered pioneers in this regard, proposed a three-factor model of adult attachment, which is explained in relation to security, anxiety, and avoidance, reflecting the infant attachment patterns that were originally observed by Ainsworth et al. (1978). Attachment security is defined as the confidence in others’ emotional availability to provide reassurance during distress and/or need. Attachment anxiety is instead characterized by a perceived inability to cope with threats and stress autonomously, which leads to the amplification of the need for interpersonal closeness (Griffin and Bartholomew, 1994). In anxiously attached individuals, these hyperactivating strategies are used to force significant others (who are perceived as not sufficiently available and responsive) to pay greater attention and provide better protection and support (Shaver and Mikulincer, 2002). Finally, attachment avoidance is characterized by the discomfort with closeness and an emphasis on emotional distance and autonomy, which is expressed through the suppression of support seeking (Bartholomew, 1990). In avoidant individuals, these deactivating strategies are used to avoid the negative emotions provoked by the attachment figures’ unavailability (Shaver and Mikulincer, 2002).

Several studies have employed self-report instruments to examine the association between parental attachment and the quality of parenting (e.g., Jones et al., 2015). These investigations have revealed that insecure attachment styles are negatively related to maternal supportiveness (Berlin et al., 2011), parental functioning (Cohen et al., 2011), and caregivers’ sensitivity (Mills-Koonce et al., 2011), particularly when parents perceive distress (Edelstein et al., 2004). It has also been documented that attachment insecurity is a risk factor for experiencing high levels of stress and arousal in the context of parent–infant relationships (Vasquez et al., 2002; Mills-Koonce et al., 2011), and that secure attachment is associated with the ability to cope with distress and adjust to parenting tasks (Alexander et al., 2001; Feeney, 2003; Jones et al., 2015; Pazzagli et al., 2015).

### Parenting Stress

Deater-Deckard (1998), who noted that individuals may experience parenting as stressful, defined parenting stress as an “aversive psychological reaction to the demands of being a parent (…) experienced as negative feelings toward the self and toward the child or children, and by definition, these negative feelings are directly attributable to the demands of parenthood” (p. 315). Abidin (1995) conceptualized parenting stress as resulting from a difficulty to adjust to the parenting role, which is influenced by parents’ perceptions of themselves as not competent, their children as problematic, and/or the relationship with children as difficult as parents. Numerous studies have employed the Parenting Stress Index–Short Form (PSI-SF; Abidin, 1995) to explore the role of parenting stress on the development of a dysfunctional parent–child relationship and have demonstrated its association with parents and children’s psychopathology (Zaidman-Zait et al., 2014; Ramsauer et al., 2016) as well as its influence on various parental characteristics, including sensitivity, dyadic pleasure, and quality of caregiving (Crnic et al., 2005; McMahon and Meins, 2012). Parenting stress has been also found to be associated with children’s sensory processing difficulties (Gourley et al., 2013) and communication difficulties (Barwick et al., 2004).

Recently, Moe et al. (2018) have demonstrated that the maternal attachment style moderates the relationship between mothers’ adverse childhood experiences and parenting stress and highlighted the relationship between perceived, infant characteristics and parenting stress.

Starting from these premises, the present study aimed to specifically examine mothers’ perceptions of their infant’s characteristics, among general population. Moreover, this study investigated mothers’ perceptions about how they behave with their children (Lee and Bates, 1985) and how their children behave with them (Schaughency and Lahey, 1985).

Because the role of parental attachment patterns has been explored in relation to parenting stress (Rholes et al., 2006) and children’s perceived characteristics (Karabekiroğlu and Rodopman-Arman, 2011), the specific aims of this study were to examine first, whether parents’ attachment styles and parenting stress predicted their perceptions of their infants’ characteristics, and, second, whether attachments styles had an effect on the relationship between parental distress and perceived infant characteristics.

- Hypothesis 1: we expected that parenting stress dimensions (that is, the stress experienced by the mothers in relation to their parental role, the interactions with the child, and the difficulties of the child, respectively) and attachment styles will be correlated with maternal perceptions of child’s characteristics (particularly, the child’s sensory-regulatory abilities).
- Hypothesis 2: we expected that attachment styles will moderate the relationship between parenting stress and perceived child’s characteristics. Specifically, we hypothesized that mothers with high levels of avoidance and/or anxiety and high levels of parenting stress will have more negative perceptions of their children’s abilities.

## Materials and Methods

### Participants and Procedures

Recruitment was carried out at maternity and child health services of Rome, where mothers had taken their 1-year-old children to undergo routine pediatric visits or vaccinations. Initially, a total of 476 mothers received the invitation to participate to the research. Of these mothers, 71 did not give their consent to be enrolled in the study, while 48 were ruled out because they did not complete all the measurements. Thus, the final samples consisted of 357 mothers (age: 34.23; ± 5.38) and their children (161 males and 196 females; age: 12.70; ± 1.60 months). The minimum sample size necessary for the intended analyses was 119 subjects. The detailed characteristics of the participants are reported in Table 1.

**TABLE 1:** Participants’ characteristics.

Variable		Frequency	Percentage%
Gender of children			
	Female	196	54.9
	Male	161	45.1
Marital status of mothers			
	Married/life partner	255	71.4
	Divorced	9	2.5
	Widowed	1	0.3
	Single	74	20.7
	Missing	18	
Nationality			
	Italian	296	82.9
	Foreign	60	16.8
	Missing	1	
Education			
	Elementary school	2	0.6
	Middle school	49	13.7
	High school	188	52.7
	Degree	109	30.5
	Missing	9	
Occupation			
	Employe	199	55.7
	Self-employed	30	8.4
	Business owner	8	2.2
	Housewife	84	23.5
	Unemployed	26	7.3
	Student	1	0.3
	Missing	9	

Prior to data collection, the mothers received complete information concerning the rationale of the study and provided their written informed consent for their participation, stated in the Declaration of Helsinki.

### Measures

#### Parenting Stress

The PSI-SF (Abidin, 1995; Italian validation by Guarino et al., 2008) is a 36-item questionnaire assessing the feelings of distress that individuals experience in relation to their role as parents. Respondents evaluate three defined latent constructs, namely, Parental Distress (PD), Parent–Child Dysfunctional Interaction (P-CDI), and Difficult Child (DC), with 12 items each on a 5-point Likert scale. The PD subscale focuses on parents’ sense of competence/incompetence in rearing their children, conflict with their partner, lack of social support, and stress associated with the restrictions experienced due to their parental role. The DC subscale examines parents’ perception of their children in relation to temperament, requesting and provoking behaviors, and non-collaborative and demanding behaviors. The P-CDI subscale measures parents’ perceptions of the emotional quality of their relationship with their children. The value of total stress is obtained by summing the scores of the three subscales, thereby indicating individuals’ overall level of stress associated with parenting, which is not a result of their other roles and/or events. The internal consistency of the Italian validation of the PSI-SF (Guarino et al., 2008) revealed α = 0.91 for the PD subscale, α = 0.95 for the P-CDI subscale, and α = 0.90 for the DC subscale.

#### Attachment Style

The ASQ (Feeney et al., 1994; Italian Validation by Fossati et al., 2003) is a 40-item self-report questionnaire that measures the adult attachment style on a 6-point Likert scale, ranging from 1 (totally disagree) to 6 (totally agree). In accordance with Pedrazza and Boccato (2010), we employed the three-factor model of this instrument that defined three latent constructs: Security or Confidence (SEC), Avoidance (AVO), and Anxiety (ANX). SEC is concerned with individuals’ sense of self-value, how easily they relate to others, the notion of the self as competent in interpersonal relationships and worthy of love, and the idea of others being available, particularly in difficult moments. AVO measures discomfort with closeness, that is, the tendency to emphasize autonomy and independence of self as well as consideration of relationships as secondary, which involves the propensity to become close to others. Finally, ANX includes individuals’ need for approval, namely, the tendency to value the self-based on others and preoccupation with relationships, that is, the need for closeness and concern about the lack of reciprocity of this need. The internal consistency of the subscales of the Italian validation (Fossati et al., 2003) found α = 0.69 for confidence, α = 0.68 for discomfort with closeness, α = 0.73 for relationships as secondary, α = 0.69 for need for approval, and α = 0.64 for preoccupation with relationships. In accordance with Pedrazza and Boccato (2010), we employed a three-factor model of the ASQ, which explained 35.7% of the variance.

#### Child Characteristics

The 1st-Year Inventory (FYI; Baranek et al., 2003; Italian Validation by Muratori et al., 2009) is a parent-report questionnaire, designed to identify 12-month-old infants at risk for autism spectrum disorder (ASD). The questionnaire comprises 63 questions. In 46 of these questions, respondents are asked to select one of the options (never, seldom, sometimes, and often) on a 4-point Likert scale. In 14 questions, respondents are required to select one of 3–4 multiple choice answers. Parents select sounds they have heard their infant utter in one question. Finally, there are two open-ended questions related to parental concerns and unusual physical and/or medical characteristics. The FYI provides a measure of children’s abilities across the social communication domain (SC Dom) and the sensory regulatory functions domain (SR Dom). The SC Dom comprises four subscales: social orienting and receptive communication, which assesses infants’ ability to focus on the object of the communication with another person; social-affective engagement, which measures infants’ ability to engage in social and affective interactions; imitation, which evaluates infants’ imitative ability; and expressive communication, which assesses infants’ abilities to get their mother’s attention or to use gestures to acquire something. SR Dom also comprises four subscales: sensory processing, which evaluates infants’ sensitivity to touch, gaze, and/or texture; regulatory patterns, which measures infants’ ability to regulate their sleeping and feeding patterns; reactivity, which assesses the difficulty of calming infants when they are upset; and repetitive behavior, which includes questions, such as whether the infant enjoys staring at a bright light for a lengthy period and whether the infant rocks back and forth continually. In this study, the FYI was not employed with the aim to detect the risk for ASD in children; Rather it was used to have an exhaustive picture of mothers’ perception of their children’s social communication and sensory regulatory abilities. The internal consistency for Social–Communication and Sensory–Regulatory Functions domains were 0.91 and 0.88, respectively (Levante et al., 2020).

### Data Analysis

First, descriptive analysis was conducted to explore the characteristics of the sample. Then, correlation analysis was performed to explore how socio-demographics, parenting stress, attachment styles and social communicative and sensory-regulatory functions correlates each other. The correlation matrix and their significative values were reported in Table 3. Multiple regression analyses were conducted with ASQ and PSI-SF as predictors of FYI child characteristics. Subsequently, a moderation analysis was performed to explore significant interaction effects among the study variables. The moderation models evaluated whether attachment styles had an effect on the relationship between PD and perceived infants’ characteristics. PROCESS SPSS 3.3 macro (Hayes, 2019) was employed to test the models. This macro determined 95% confidence intervals in relation to the attachment scores at which the effect of the PD was significantly associated with perceived children’s characteristics. Significant interactions were subsequently examined graphically to assess regression slopes of parenting stress on perceived children’s characteristics at low (i.e., 1 SD below the mean) and high (1 SD above the mean) values of the ASQ. Beyond statistical significance, interaction effects were also appraised in relation to effect size (ΔR2). As recommended by Hair et al. (2017), 0.02, 0.15, and 0.35 represent small, medium, and large effect sizes, respectively.

## Results

Table 2 presents means and standard deviations for the scores on the three subscales of PSI-SF, three subscales of ASQ, and two domains of the FYI.

**TABLE 2:** Summary of Means and Standard Deviations for scores on PSI-SF subscales, ASQ dimensions and FYI domains.

Variables	Mean	SD	Cronbach’s α
(1) PD	25.18	8.14	0.87
(2) P-CDI	18.82	4.78	0.76
(3) DC	21.26	5.71	0.77
(4) SEC	38.67	4.53	0.63
(5) AVO	22.25	6.17	0.82
(6) ANX	21.23	5.66	0.83
(7) SC Dom	2.87	3.10	0.61
(8) SR Dom	5.25	4.94	0.71

*PD, Parental Distress (PSI-SF); P-CDI, Parent–Child Dysfunctional Interaction (PSI-SF); DC, Difficult Child (PSI-SF); SEC, Security (ASQ); AVO, Avoidance (ASQ); ANX, Anxiety (ASQ); SC Dom, Social Communication Domain (FYI); and SR Dom, Sensory Regulatory Functions Domain (FYI).*

Table 3 shows that the internal consistencies of the scales measured using Cronbach’s α coefficients ranged from acceptable (0.60–0.70) to very good (>0.80) (Hulin et al., 2001; Ursachi et al., 2015). This preliminary test was necessary to perform the following analyses.

**TABLE 3:** Correlation matrix.

		Sex Child	Mother’s Nationality	Mother’s Education	Mother’s marital status	Mother’s Occupation	Birth order	Child Age	Child weight	Number of children	Mother’s Age	SC	FR	SEC	ANX	AVO	PD	P-CDI	DC
												Dom	Dom						
	coefficient	1,000																	
Sex Child																			
	*p*-value																		
Mother’s Nationality	coefficient	5.336*	1,000																
	*p*-value	**0.021**																	
Mother’s Education	coefficient	0.151	15.682**	1,000															
	*p-*value	0.985	**0.001**	.															
Mother’s marital status	coefficient	4.513	0.291	14.579	1,000														
	*p*-value	0.211	0.962	0.103															
Mother’s Occupation	coefficient	13.296*	67.574**	88402**	15.674	1,000													
	*p*-value	**0.021**	**0.000**	**0.000**	0.404														
Birth order	coefficient	0.021	0.016	−0.153**	0.171*	0.131	1.000												
	*p-*value	0.661	0.735	**0.001**	**0.011**	0.187													
Child Age	coefficient	0.026	0.009	**−**0.006	0.074	0.105	0.077	1.000											
	*p*-value	0.571	0.843	0.889	0.533	0.404	0.104												
Child weight	coefficient	0.177**	0.036	0.001	0.066	0.147	0.030	0.015	1.000										
	*p-*value	**0.000**	0.444	0.987	0.635	0.079	0.534	0.754											
Number of children	coefficient	0.024	0.000	−0.165*	0.171*	0.145	0.967**	0.096*	**−**0.023	1.000									
	*p*-value	0.606	0.994	**0.001**	**0.012**	0.102	**0.000**	**0.044**	0.634										
Mother’s Age	coefficient	0.093*	0.288**	0.118*	0.126	0.193**	0.212**	0.026	**−**0.059	0.218**	1.000								
	*p*-value	**0.049**	**0.000**	**0.013**	0.098	**0.005**	**0.000**	0.579	0.214	**0.000**									
SC Dom	coefficient	0.053	0.067	−0.135**	0.078	0.174*	**−**0.003	**−**0.014	0.011	0.029	**−**0.058	1.000							
	*p*-value	0.291	0.183	**0.007**	0.516	**0.034**	0.957	0.784	0.830	0.574	0.251								
FR Dom	coefficient	0.044	0.255**	−0.225**	0.059	0.282**	**−**0.030	**−**0.020	**−**0.085	**−**0.003	**−**0.086	0.133**	1.000						
	*p*-value	0.378	**0.000**	**0.000**	0.724	**0.000**	0.558	0.683	0.090	0.949	0.092	**0.007**							
SEC	coefficient	0.058	0.187**	0.052	0.061	0.155	0.098	−0.120*	**−**0.013	0.077	0.129*	−0.124*	−0.144**	1.000					
	*p*-value	0.279	**0.000**	0.331	0.741	0.141	0.072	**0.024**	0.816	0.162	**0.016**	**0.019**	**0.007**						
ANX	coefficient	0.008	0.248**	−0.107*	0.050	0.205*	**−**0.103	0.057	**−**0.046	**−**0.080	−0.157**	0.087	0.301**	−0.433**	1.000				
	*p*-value	0.880	**0.000**	**0.047**	0.838	**0.012**	0.062	0.282	0.393	0.147	**0.003**	0.101	**0.000**	**0.000**					
AVO	coefficient	0.041	0.379**	−0.191**	0.119	0.246**	**−**0.079	**−**0.046	**−**0.030	**−**0.034	−0.250**	0.130*	0.273**	−0.417**	0.622**	1.000			
	*p*-value	0.445	**0.000**	**0**	0.196	**0.001**	**0.149**	0.384	0.575	0.541	**0.000**	**0.015**	**0.000**	**0.000**	**0.000**				
PD	coefficient	0.080	0.146**	**−**0.041	0.193**	0.216**	**−**0.049	0.048	**−**0.043	**−**0.023	0.015	0.128*	0.299**	−0.383**	0.489**	0.381**	1.000		
	*p*-value	0.112	**0.003**	0.415	**0.003**	**0.003**	0.346	0.338	0.396	0.662	0.764	**0.011**	**0.000**	**0.000**	**0.000**	**0.000**			
P-CDI	coefficient	0.110*	0.302**	−0.164**	0.100	0.203**	**−**0.058	0.028	**−**0.012	**−**0.036	**−**0.098	0.174**	0.345**	−0.292**	0.269**	0.325**	0.463**	1.000	
	*p*-value	**0.028**	**0.000**	**0.001**	0.292	**0.006**	0.261	0.584	0.812	0.490	0.056	**0.000**	**0.000**	**0.000**	**0.000**	**0.000**	**0.000**		
DC	coefficient	0.075	0.255**	−0.108*	0.015	0.148	**−**0.070	0.092	0.011	**−**0.035	**−**0.022	0.110*	0.434**	−0.209**	0.376**	0.304**	0.460**	0.563**	1.000
	*p*-value	0.134	**0.000**	**0.033**	0.993	0.128	0.177	0.066	0.834	0.501	0.664	**0.028**	**0.000**	**0.000**	**0.000**	**0.000**	**0.000**	**0.000**	

*The indices presented are of different nature: the associations between nominal variables and continuous variables were measured with Eta index; the associations between ordinal variables and continuous variables were measured with Spearman index; the associations between continuous variables were measured with Pearson index; associations between nominal variables were measured with chi-square. Bold values are significant values (*p* < 0.05).*

First, we examined how the attachment styles and parenting stress predicted mothers’ perception of their infants’ characteristics in the SC Dom and SR Dom.

As displayed in Table 4 (panels a, b, and c), P-CDI was the only aspect of parenting stress that predicted infants SC Dom in mothers’ perceptions. Attachment styles did not predict these perceived abilities. The regression model accounted for 40% of the variance of SC Dom characteristics.

**TABLE 4:** Summary of hierarchical regression analysis for parenting stress and attachment styles to predict children’s social communication abilities.

**Panel (a)**					

	**Tests of Model Coefficients**				
	**B**	**SE(B)**	**Beta**	***t*-value**	**Sign.**

Step 1 (*R*^2^ = 0.01; *F* = 3.79)					
Parenting Distress (PD)	0.039	0.020	0.103	1.947	0.058
Step 2 (*R*^2^ = 03; *F* = 2.22)					
Parenting Distress (PD)	0.025	0.023	0.065	1.056	0.300
Security (SEC)	−0.059	0.041	−0.087	−1.422	0.146
Avoidance (AVO)	0.047	0.034	0.095	1.408	0.218
Anxiey (ANX)	−0.034	0.039	−0.063	−0.887	0.386

**Panel (b)**					

	**Tests of Model Coefficients**				
	**B**	**SE(B)**	**Beta**	***t*-value**	**Sign.**

Step 1 (*R*^2^ = 0.03; *F* = 10.52)					
Parent-Child Dysfunctional Interaction (P-CDI)	0.109	0.034	0.170	3.24	0.001
Step 2 (*R*^2^ = 0.04; *F* = 3.50)					
Parent-Child Dysfunctional Interaction (P-CDI)	0.089	0.036	0.139	2.472	,010
Security (SEC)	−0.052	0.040	−0.077	−1.287	0.282
Avoidance (AVO)	0.035	0.034	0.071	1.050	0.351
Anxiey (ANX)	−0.028	0.037	−0.052	−0.769	0.546

**Panel (c)**					

	**Tests of Model Coefficients**				
	**B**	**SE(B)**	**Beta**	***t*-value**	**Sign.**

Step 1 (*R*^2^ = 0.01; *F* = 2.15)					
Difficult Child (DC)	0.042	0.028	0.078	1.466	0.160
Step 2 (*R*^2^ = 02; *F* = 2.13)					
Difficult Child (DC)	0.026	0.031	0.049	−0.859	0.412
Security (SEC)	−0.066	0.040	−0.098	−1.641	0.111
Avoidance (AVO)	0.047	0.034	−0.094	1.394	0.191
Anxiey (ANX)	0.030	0.038	−0.054	−0.778	0.448

*N = 355; PD, Parenting Distress (PSI-SF); P-CDI, Parent–Child Dysfunctional Interaction (PSI-SF); DC, Difficult Child: (PSI-SF); SEC, Security (ASQ); AVO, Avoidance (ASQ); ANX, Anxiety (ASQ); SC Dom, Social Communication Domain (FYI); and SR Dom, Sensory Regulatory Functions Domain (FYI).*

When predicting the perceived SR Dom (Table 5), all the dimensions of parenting stress were significantly associated with the dependent variable. It is noteworthy that the attachment styles, particularly anxious attachment, contributed significantly to a biased perception of SR Dom abilities controlled for parenting stress. The regression models accounted for 14%, 19%, and 23% of the variance of SR Dom characteristics, respectively.

**TABLE 5:** Summary of hierarchical regression analysis for parenting stress and attachment styles to predict children’s regulatory functions.

**Panel (a)**					

	**Tests of Model Coefficients**				
	**B**	**SE(B)**	**Beta**	***t*-value**	**Sign.**

Step 1 (*R*^2^ = 0.09; *F* = 35.81)					
Parenting Distress (PD)	0.185	0.031	0.303	5.984	0.000
Step 2 (*R*^2^ = 0.14; *F* = 14.21)					
Parenting Distress (PD)	0.121	0.035	0.198	3.405	0.001
Security (SEC)	0.059	0.063	0.054	0.947	0.468
Avoidance (AVO)	0.108	0.051	0.135	2.124	0.088
Anxiey (ANX)	0.141	0.059	0.159	2.387	0.016

**Panel (b)**					

	**Tests of Model Coefficients**				
	**B**	**SE(B)**	**Beta**	***t*-value**	**Sign.**

Step 1 (*R*^2^ = 0.13; *F* = 52.89)					
Parent-Child Dysfunctional Interaction (P-CDI)	0.373	0.051	0.361	7.272	0.000
Step 2 (*R*^2^ = 0.19; *F* = 19.95)					
Parent-Child Dysfunctional Interaction (P-CDI)	0.303	0.054	0.294	5.656	0.011
Security (SEC)	0.068	0.060	0.062	1.124	0.305
Avoidance (AVO)	0.072	0.050	0.089	1.424	0.350
Anxiey (ANX)	0.180	0.055	0.204	3.296	0.001

**Panel (c)**					

	**Tests of Model Coefficients**				
	**B**	**SE(B)**	**Beta**	***t*-value**	**Sign.**

Step 1 (*R*^2^ = 0.19; *F* = 84.50)					
Difficult Child (DC)	0.381	0.042	0.439	9.192	0.000
Step 2 (*R*^2^ = 0.23; *F* = 25.54)					
Difficult Child (DC)	0.319	0.044	0.367	7.203	0.000
Security (SEC)	0.032	0.058	0.029	0.548	0.741
Avoidance (AVO)	0.087	0.048	0.108	1.800	0.161
Anxiey (ANX)	0.109	0.055	0.123	1.985	0.042

*N = 355; PD, Parental Distress (PSI-SF); P-CDI, Parent–Child Dysfunctional Interaction (PSI-SF); DC, Difficult Child (PSI-SF); SEC, Security (ASQ); AVO, Avoidance (ASQ); ANX, Anxiety (ASQ); SC Dom, Social Communication Domain (FYI); and SR Dom, Sensory Regulatory Functions Domain (FYI).*

Moderation analyses were conducted to examine the interactions of PD and attachment styles as predictors of child characteristics.

**FIGURE 1:**
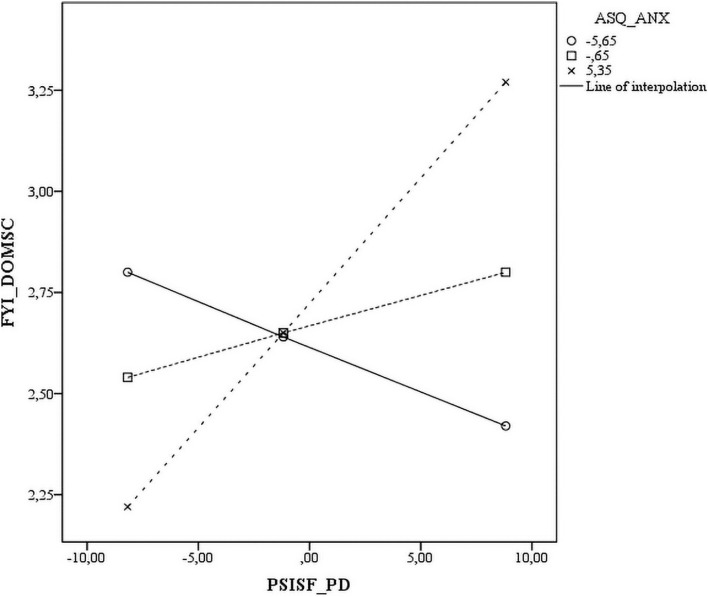
Scatter plot of the interaction between anxious attachment style and parental distress on the socio communicative domain.

**FIGURE 2:**
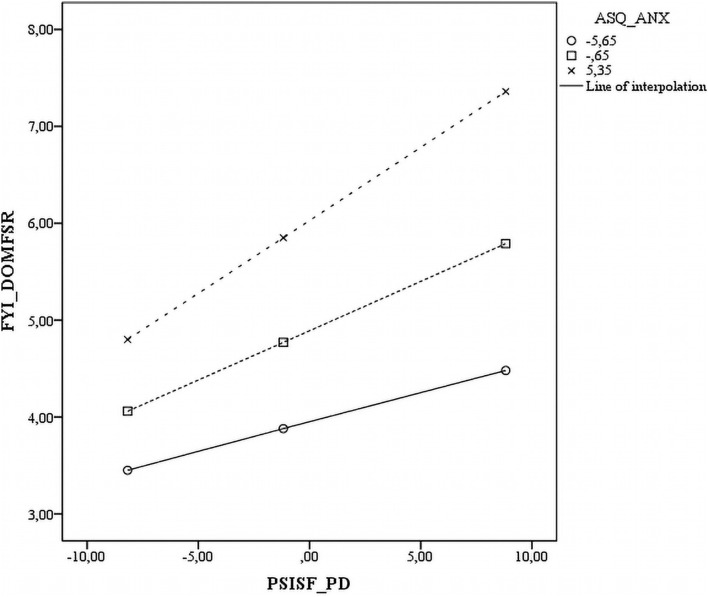
Scatter plot of the interaction between anxious attachment style and parental distress on the sensory regulation domain.

**FIGURE 3:**
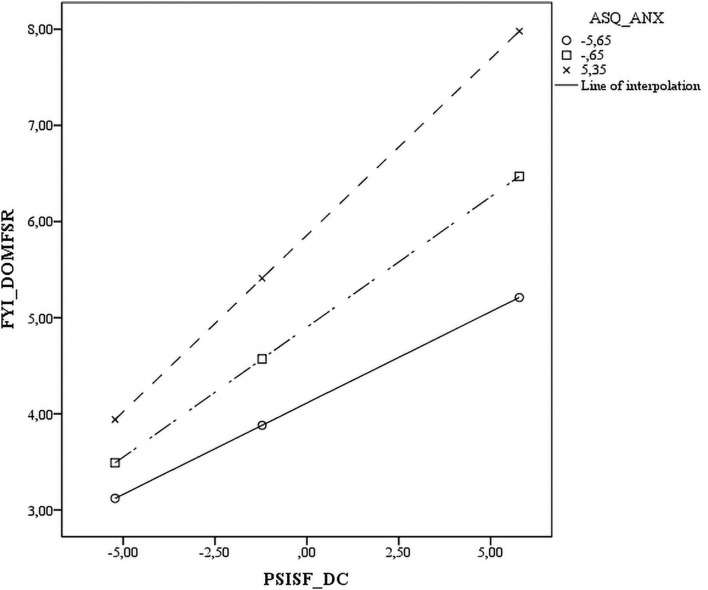
Scatter plot of the interaction between anxious attachment style and difficult child on the sensory regulation domain.

**FIGURE 4:**
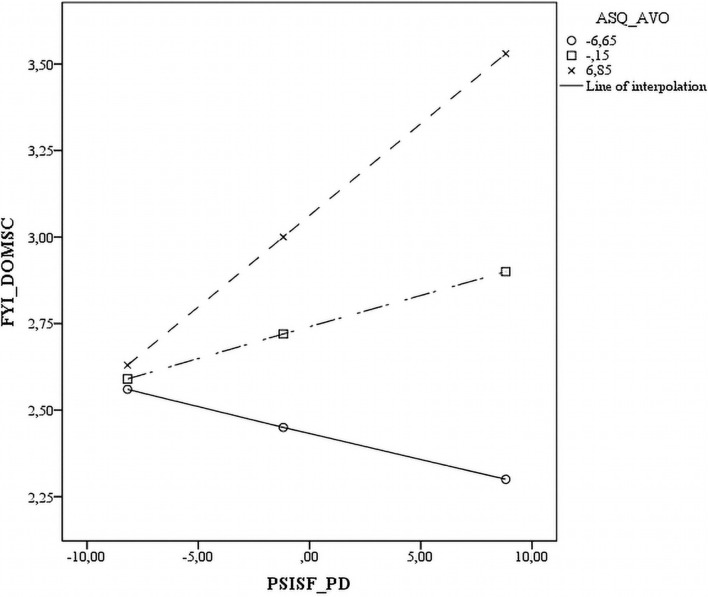
Scatter plot of the interaction between avoidant attachment style and parental distress on the socio communicative domain.

**FIGURE 5:**
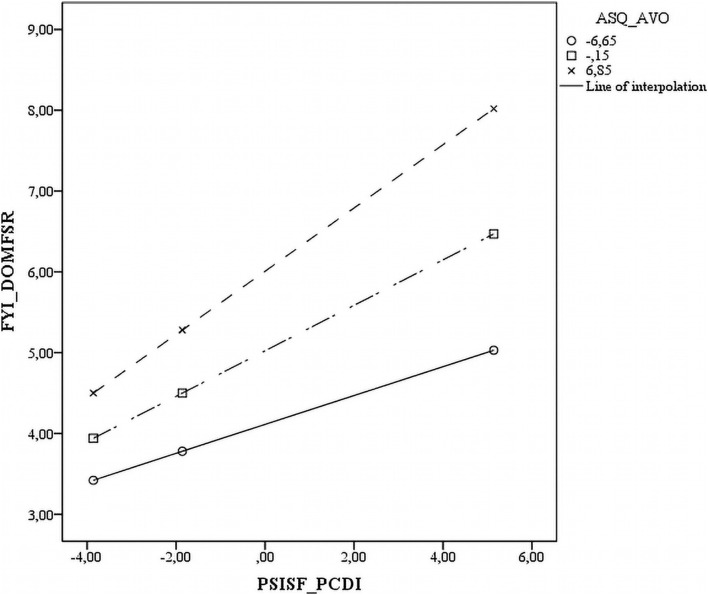
Scatter plot of the interaction between anxious attachment style and parent-child dysfunctional interaction on the socio communicative domain on the sensory regulation domain.

Five of the interactions tested between the three ASQ subscales and three PSI-SF subscales were statistically significant across all measures. The significant interactions are plotted in Figures 1–5. The results revealed that specific mothers’ attachment styles moderated the association between specific dimensions of parenting stress and their perception of their infants’ characteristics.

In particular, the PD results that were employed to predict the perception of the infants’ SC Dom were dependent on mothers’ levels of avoidance. In mothers with low levels of AVO (-1 SD), the greater the PD, the less the SC Dom, whereas in mothers with high levels of AVO (+1 SD), the greater the PD, the greater the SC Dom (Figure 1).

The P-CDI results that were used to predict SR Dom were also dependent on mothers’ levels of avoidance. In those with low levels of AVO (-1 SD) and high levels of AVO (+ 1 SD), the greater the P-CDI, the greater the SR Dom. However, the levels of P-CDI were significantly higher in those with high AVO than in those with low AVO (Figure 2).

A similar pattern was found for anxious attachment styles. As noted previously, PD predicted SC abilities. However, in mothers with low levels of ANX (-1 SD), the greater the PD, the less the SC Dom was, whereas in mothers with high levels of ANX (+ 1 SD), the greater the PD, the greater the SC Dom (Figure 3).

Parenting distress also predicted SR functions. However, mothers’ anxiety levels influenced this relation differently. In those with low ANX (-1 SD) and high ANX (+ 1 SD), the greater the PD, the greater the SR Dom. However, the levels of PD were significantly higher in mothers with high ANX than in those with low ANX (Figure 4).

Finally, the DC results were also employed to predict SR functions. In mothers with low ANX (-1 SD) and high ANX (+ 1 SD), the greater the DC, the greater the SR Dom. However, the DC levels were significantly higher in those with high ANX than in those with low ANX (Figure 5).

## Discussion

As we highlighted in the introduction of this paper, research has shown an association between parental attachment and perception of children’s characteristics (Karabekiroğlu and Rodopman-Arman, 2011) and between parenting stress and children’s abilities (Barwick et al., 2004; Gourley et al., 2013). Accordingly, the purposes of this study were to explore how attachment styles and parenting stress contribute to the prediction of mothers’ perceptions of their infant’s characteristics in the SC and SR Dom. Moreover, this study aimed to examine whether attachments styles had an effect on the relationship between parental distress and perceived infant characteristics.

Regression analyses confirmed that parenting stress predicted mothers’ perceptions of children’s characteristics. The results further revealed that the incremental value of attachment styles was significant, even if only for the prediction of the SR Dom. Regression analyses with attachment styles was performed in the second step. In particular, even if children’s abilities in the SR Dom were predicted by mothers’ perception of experiencing stress because of their PD, the mothers’ levels of avoidant (AVO) and anxious (ANX) attachment styles contributed to their perception of children’s abilities in this field: mothers who had more insecure attachment styles also reported higher levels of PD. Similar results were found after considering that the perception of children’s SR Dom were influenced by mothers’ perspective of how difficult the interaction was (P-CDI) and how many of these difficulties were caused by the children’s characteristics (DC). In both cases, mothers’ anxiety contributed substantially to a more negative perception of the children’s characteristics when controlled for parenting stress dimensions. Our Hypothesis 1 was therefore partially confirmed. This result concurs with research on the role of insecure attachment in parenting (George and Solomon, 1996, 1999). Moreover, it highlights that attachment style has a specific role in mothers’ perception of their children’s characteristics even if the contribution of parenting stress is considered, which is subsequently discussed.

Based on evidence that parental attachment patterns can influence parenting stress (Rholes et al., 2006), we explored the role of attachment style as a moderator in the association between parenting stress and mothers’ perceptions of children’s SC and SR Dom, considering that these relationships differ substantially and rely on mothers’ own adult attachment styles. The results revealed that the relationship between parenting stress and perceived children’s characteristics was moderated by the attachment style, as we stated in our Hypothesis 2. Specifically, when levels of avoidance were very high and a mother perceived that her parenting role was stressful (high PD), she viewed her child as considerably less able in the social communication field than if she had low levels of PD. By contrast, when levels of avoidance were low, mothers with high PD perceived their children as less difficult in the SC Dom than those with low levels of PD. Therefore, a low level of avoidance was a protecting factor for mothers who experienced their parenting role as a source of distress. Moreover, high avoidance levels influenced how mothers who considered the interaction with their children as difficult perceived them as having greater difficulties in relation to SR Dom. By contrast, when levels of anxiety were very high and mothers perceived that their parenting role was stressful, they believed their children were less able in the SC Dom than if they had low levels of PD. When mothers’ levels of anxiety were very low, those with high PD viewed their children as less difficult in the SC Dom in comparison to those with low levels of PD. This evidence confirms the protecting role of low levels of anxiety when mothers experience their parental role as stressful and subsequently perceive their children as difficult.

Finally, anxiety levels also had a different influence on mothers who considered their parental role as stressful or perceived their children as difficult.

Although this result further confirmed the importance of parental attachment style as a fundamental protective factor in the parenting process, it was more evident in the interaction between parents and children.

To the best of our knowledge, the specific moderating role of different levels of insecure attachment styles in the relationship between parenting stress and perceived children’s characteristics is a new contribution to the literature, even if linked to the role of attachment in overall parental sensitivity (Paulussen-Hoogeboom et al., 2008).

## Limitations and Future Directions

The current study has several constraints. First, it was a cross-sectional study with no external criteria. Thus, the direction of causality was explained in accordance with the literature. Second, the mothers evaluated both their own parenting stress and their children’s characteristics, thereby possibly resulting in an evaluation bias. It is recommended that longitudinal studies using self and informant ratings, such as those of pediatricians, be conducted to establish causal relationships and cross-validate parents’ perceptions.

A further limitation concerns statistical analyses. In fact, we performed as many regressions as there were dimensions of parenting stress, by differentiating each one but not considering the simultaneous role played by all of them in influencing the infants’ social communication and sensory regulatory functions.

Notwithstanding these limitations, we believe that the present study has shed light on how parenting stress can influence how mothers with different attachment styles perceive their children’s behavior, particularly in relation to social communication abilities and sensory regulatory functions.

We also believe that our study has significant clinical implications, guiding the prevention programs that could be addressed to mothers with insecure attachment style, with the aim to sustain their ability to meet demands of parenting and – as a result – to adequately detect their children’s sensory regulation and communication competencies.

## Data Availability Statement

The data analyzed in this study is subject to the following licenses/restrictions: the data presented is part of a larger research with a follow-up that has not yet been published. Requests to access these datasets should be directed to MQ, maria.quintigliano@uniroma1.it.

## Ethics Statement

The studies involving human participants were reviewed and approved by Department of Dynamic and Clinical Psychology, and Health Studies Sapienza University of Rome. The patients/participants provided their written informed consent to participate in this study.

## Author Contributions

AS and CT planned and developed the study design. MQ, CT, AF, ML, and AS all contributed in planning of the design of the present manuscript. MQ and ML performed analysis of data. MQ took the main responsibility for drafting the article, while CT, AF, and AS contributed substantially in revising it critically for important intellectual content. All authors approved the final draft for publication.

## Conflict of Interest

The authors declare that the research was conducted in the absence of any commercial or financial relationships that could be construed as a potential conflict of interest.

## Publisher’s Note

All claims expressed in this article are solely those of the authors and do not necessarily represent those of their affiliated organizations, or those of the publisher, the editors and the reviewers. Any product that may be evaluated in this article, or claim that may be made by its manufacturer, is not guaranteed or endorsed by the publisher.
